# Pharmacologic targeting of the P-TEFb complex as a therapeutic strategy for chronic myeloid leukemia

**DOI:** 10.1186/s12964-021-00764-5

**Published:** 2021-08-09

**Authors:** Yingjie Qing, Xiangyuan Wang, Hongzheng Wang, Po Hu, Hui Li, Xiaoxuan Yu, Mengyuan Zhu, Zhanyu Wang, Yu Zhu, Jingyan Xu, Qinglong Guo, Hui Hui

**Affiliations:** 1 State Key Laboratory of Natural Medicines, Jiangsu Key Laboratory of Carcinogenesis and Intervention, China Pharmaceutical University, 24 Tongjiaxiang, Nanjing, 210009 People’s Republic of China; 2 Department of Pharmacology, School of Medicine and Holostic Integrative Medicine, Nanjing University of Chinese Medicine, Nanjing, 210023 People’s Republic of China; 3 Department of Hematology, The First Affiliated Hospital of Nanjing Medical University, Jiangsu Province Hospital, Nanjing, 210029 People’s Republic of China; 4 Department of Hematology, The Affiliated DrumTower Hospital of Nanjing University Medical School, Nanjing, 210008 People’s Republic of China

**Keywords:** Wogonin, Cell differentiation, P-TEFb, CDK9, CML

## Abstract

**Background:**

The positive transcription elongation factor b (P-TEFb) kinase activity is involved in the process of transcription. Cyclin-dependent kinase 9 (CDK9), a core component of P-TEFb, regulates the process of transcription elongation, which is associated with differentiation and apoptosis in many cancer types. Wogonin, a natural CDK9 inhibitor isolated from *Scutellaria baicalensis.* This study aimed to investigate the involved molecular mechanisms of wogonin on anti- chronic myeloid leukemia (CML) cells.

**Materials and methods:**

mRNA and protein levels were analysed by RT-qPCR and western blot. Flow cytometry was used to assess cell differentiation and apoptosis. Cell transfection, immunofluorescence analysis and co-immunoprecipitation (co-IP) assays were applied to address the potential regulatory mechanism of wogonin. KU-812 cells xenograft *NOD/SCID* mice model was used to assess and verify the mechanism in vivo.

**Results:**

We reported that the anti-CML effects in K562, KU-812 and primary CML cells induced by wogonin were regulated by P-TEFb complex. We also confirmed the relationship between CDK9 and erythroid differentiation via knockdown the expression of CDK9. For further study the mechanism of erythroid differentiation induced by wogonin, co-IP experiments were used to demonstrate that wogonin increased the binding between GATA-1 and FOG-1 but decreased the binding between GATA-1 and RUNX1, which were depended on P-TEFb. Also, wogonin induced apoptosis and decreased the mRNA and protein levels of MCL-1 in KU-812 cells, which is the downstream of P-TEFb. In vivo studies showed wogonin had good anti-tumor effects in KU-812 xenografts *NOD/ SCID* mice model and decreased the proportion of human CD45^+^ cells in spleens of mice. We also verified that wogonin exhibited anti-CML effects through modulating P-TEFb activity in vivo.

**Conclusions:**

Our study indicated a special mechanism involving the regulation of P-TEFb kinase activity in CML cells, providing evidences for further application of wogonin in CML clinical treatment.

**Video Abstract**

**Supplementary Information:**

The online version contains supplementary material available at 10.1186/s12964-021-00764-5.

## Background

CML is a malignant clonal disease that originates in hematopoietic stem cells [1]. Clinically, it is divided into three phases including chronic phase, accelerated phase and blast phase [2]. CML patients commonly arise t(9;22)(q34;11) chromosomal translocation and formation of fusion gene *BCR-ABL1*, which is transcribed and translated into BCR-ABL1 protein with tyrosine kinase activity, thereby activating downstream signaling pathways such as PI3K-Akt-mTOR and MAPK, leading to abnormal cell proliferation [1, 3, 4]. Tyrosine kinase inhibitors (TKIs) are currently the preferred drug in clinical CML patients and Imatinib is the first generation of TKIs used for the treatment of CML [5]. However, although TKIs therapy can alleviate the condition of most CML patients, long-term use cannot eliminate the existence of factors such as leukemia stem cells and high price [6, 7]. Therefore, new ideas should be expanded in the development of CML drugs to solve the increasing urgent problems of clinical medication of CML.

Cyclin-dependent kinases (CDKs) are serine/threonine kinases found in yeast, which have the functions of regulating cell cycle and cell transcription [8, 9]. Cycle CDKs (CDK1, 2, 4, 6) mainly play a role in the regulation of cell cycle, while transcriptional CDKs (CDK7-9, 10–13) act on the transcriptional regulation process of cells [10].Transcription CDKs make RNA Polymerase II (RNA Pol II) carboxyl terminal structure domain (C-Terminal Domain, CTD) phosphorylation, thus promote the initiation and extension of transcription [11]. Inhibition of CDKs mainly affects the accumulation of transcripts with short half-life, such as anti-apoptotic family members' genes *BCL2*, *MCL1* and *XIAP*, cell cycle regulating family genes *CCND1* and *CMYC*, and tumor suppressor genes *TP53* [10]. Therefore, transcriptional CDKs is one of the potential targets of tumor therapy.

RNA Pol II is a transcription enzyme, which regulates the messenger RNA and non-coding RNA [12]. RNA pol II activity in the transcription cycle stages is strictly regulated, which makes phosphorylation RNA pol II CTD in dynamic changes and drives transcription from pre-initiation, initiation, extension to termination [13]. RNA transcription is catalyzed by CDK7 and CDK9 in turn. Firstly, CDK7 phosphorylates the serine (ser5) site of CTD of RNA pol II, thus activating RNA pol II and acting as a part of pre-transcriptional initiation complex to promote the initiation of transcription [14]. Subsequently, the serine (ser2) at the second position of CTD of RNA pol II is phosphorylated by CDK9, which promotes the transcription process into the extension stage [15, 16]. P-TEFb is a kinase complex formed by catalytic subunit CDK9 and regulatory subunit cyclin T1. Under the action of catalytic subunit CDK9, ser2 of CTD of RNA pol II is phosphorylated, which promotes RNA transcription extension [17]. Therefore, P-TEFb plays an important role in the production of cellular mRNA. CDK9 inhibitors inhibit the transcription of downstream genes by reducing the phosphorylation of RNA pol II by inhibiting the formation of P-TEFb [15]. Therefore, targeting CDK9 is an effective tumor therapy strategy [18].

Differentiation induction therapy is a treatment method that uses differentiation inducers to induce malignant tumor cells to transform into mature cells. Normal cells are less affected by differentiation inducers due to their high degree of differentiation. Therefore, compared with chemotherapeutic drugs with poor selectivity and high cytotoxicity, differentiation inducers have lower side effects [19]. Differentiation induction therapy has achieved good effects in the treatment of acute promyelocytic leukemia (APL), which is a model of cell differentiation therapy [20]. However, only retinoic acid (RA) combined with arsenic trioxide has been successful in the treatment of APL. Thus, new differentiation inducers are needed to develop in the treatment of CML.

During the process of hematopoiesis, hematopoietic stem cells (HSC) first differentiate into common myeloid progenitor (CMP), and then CMP differentiate into megakaryocyte erythrocyte progenitor (MEP) and granulocyte macrophage progenitor (GRA) cells. However, MEP can further differentiate into primordial megakaryocytes and erythrocytes, and then they enter the differentiation pathways of megakaryocytes and erythrocytes respectively [21]. The differentiation of MEP into megakaryocytes and erythrocytes is affected by several key transcription factors, such as GATA binding protein 1 (GATA-1), friend of GATA-1 (FOG-1), Runt-related transcription factor 1 (RUNX1), etc. [22]. GATA-1 is a transcription factor that can initiate erythroid / megakaryocyte differentiation. When MEP cells are at the transition point of erythroid / megakaryocyte differentiation, GATA-1 is activated [23]. The selection of GATA-1 for the direction of cell differentiation is related to the active complex formed by GATA-1 and other regulatory proteins. GATA-1 interacts with the N-terminal zinc finger structure of FOG-1 to activate the expression of erythroid related gene γ-globin and promote erythroid differentiation of cells [24]. On the contrary, GATA-1 forms a complex with RUNX1, which acts on the promoter GPIbα of megakaryocytes and promotes the differentiation of megakaryocytes, and the process of differentiation highly dependents on P-TEFb [25]. Thus, P-TEFb plays an important role in megakaryocyte/erythroid transition point and is the key regulatory molecule of directional differentiation. Inhibition of CDK9 kinase activity can inhibit the activity of P-TEFb, weaken the interaction between GATA-1 and RUNX1 protein, and inhibit megakaryocyte differentiation, thus promoting erythroid differentiation of cells.

Wogonin (5,7-dihydroxy-8-methoxy-2-phenyl-4h-1-benzopyran-4-one) is one of the bioactive components isolated from the Chinese herbal medicine *Scutellaria baicalensis*, which has anti-oxidant, anti-cancer, anti-inflammatory and other pharmacological activities [26, 27]. Here, we investigated P-TEFb kinase activity in regulating differentiation in CML cells. Moreover, we verified that anti-proliferation activity of wogonin involved in P-TEFb in vitro. Finally, we established a *NOD/SCID* mouse model bearing KU-812 tumors to further verify the anticancer effect and potential mechanism of wogonin.

## Materials and methods

### Compounds and regents

Wogonin (Purity > 99%) was isolated from *Scutellaria Baicalensis* according to previously reported method [28]. Wogonin was dissolved in dimethyl sulfoxide (DMSO, Sigma-Aldrich, Missouri, USA) at a concentration of 0.1 M and stored at −80 °C. Then the solution was diluted to the indicated concentration with RPMI-1640 or IMDM medium (GIBCO, CarlsBAD, CA, USA). FITC anti-human CD71 (Transferrin Receptor), PE anti-human Glycophorin A (CD235a), FITC anti-human CD41 and PE anti-human CD61 antibodies were purchased from eBioscience (San Diego, CA, USA).

For in vivo experiments, wogonin was prepared as an intraperitoneal injection administration formulation by Dr. Xue Ke at the College of Pharmacy, China Pharmaceutical University. Imatinib Mesylate was purchased from CHIA TAI TIANQING PHARMACEUTICAL GROUP CO.LTD (Nanjing, China) and prepared as an intragastric administration.

### Cell cultures

The chronic myeloid leukemia cell lines K562 and KU-812, human embryonic kidney cell line 293 T were purchased from the Cell Bank of the Shanghai Institute of Biochemistry & Cell Biology. Primary chronic myeloid leukemia cells from newly diagnosed CML patients (The Affiliated DrumTower Hospital, Nanjing, China) were collected using lymphocyte-monocyte separation medium (Jingmei, Nanjing, China). K562 cells and primary CML cells were cultured in RPMI-1640 medium, KU-812 cells were cultured in IMDM medium, 293 T cells were cultured in DMEM medium, supplemented with 10% fetal bovine serum (FBS) (GIBCO, CarlsBAD, USA), 100 U/ml of benzylpenicillin and 100 U/ml of streptomycin in a humidified environment with 5% CO_2_ at 37 °C.

### Flow cytometric analysis of apoptosis

Annexin V/PI staining assay was used to detect apoptosis of cells after wogonin treatment. The cells were collected and stained with Annexin V/PI Cell Apoptosis Detection Kit (Vazyme biotec, Nanjing, China) according to the protocols. The fluorescence was detected by flow cytometry (Becton–Dickinson Biosciences, New Jersey, USA). and the data analysis was performed by Flowjo software.

### Flow cytometric analysis of cell differentiation

K562 and KU-812 cells with different concentrations of wogonin were collected, the residual medium was washed with PBS, 0.5% BSA solution was added and blown evenly. The non-specific binding sites on the surface of the two cells were sealed, centrifuged and washed with PBS to remove the residual BSA solution. After dispersing the cell mass with 50 μL PBS, a certain amount of antibody was added to fully bind the fluorescently labeled antibody to the differentiated antigen on the CML cell surface. The antibody was incubated in a 37 °C shaker in darkness for 50 min. Unbound antibody was washed with PBS and centrifuged. After resuspended with 400 μL PBS, the positive cell percentage was analyzed and calculated using Flowjo program.

### Western blot analysis

Total proteins were extracted from cells after wogonin treatments for indicated time. The cells were lysed in RIPA buffer (Thermo Scientific, Massachusetts, USA) with protease inhibitors (PMSF, NaF, leupeptin, and dithiothreitol) on ice for 50 min and then cell lysates were clarified by centrifugation at 14,000 rpm (Eppendorf, Hamburg, Germany) for 20 min at 4 °C. The concentration of proteins was quantified with a BCA Protein Assay Kit (Thermo Fisher Scientific, USA). Equal amounts of proteins were separated with 8–12% SDS-PAGE and transferred to the PVDF membranes (Millipore, Boston, MA, USA). Membranes were blocked with 3% BSA and incubated overnight at 4 °C with primary antibodies, followed by incubation with HRP-coupled secondary antibody for 1 h at room temperature. After washing the membranes with PBST three times, detection was performed by AI600 imaging system (GE Healthcare, Pittsburgh, PA, USA).

Primary antibodies specific for RNA Pol II, p-RNA Pol II -Ser2, p-RNA Pol II -Ser5, MCL-1, Cyclin T1, FOG-1, GAPDH and β-actin were obtained from ABclonal Technology (Wuhan, China). Antibodies against CDK9, GATA-1 were obtained from Cell Signaling Technology (Danvers, MA). Antibody against RUNX1 was obtained from Proteintech (Wuhan, China). HRP Goat Anti-Rabbit IgG (H + L) and HRP Goat Anti-Mouse IgG (H + L) secondary antibody were obtained from ABclonal Technology (Wuhan, China).

### Immunoprecipitation

Cell lysate was incubated with special antibody and 20 µL protein A/G-conjugated beads (MCE) at 4 °C overnight. After washing three times with PBS buffer, samples were collected and re-suspended in 20 µL SDS-sample buffer (0.5 M Tris–HCl, pH 6.8, 20% glycerol, 2% SDS, 5% 2-mercaptoethanol and 4‰ bromophenol blue) and boiled for 10 min. Then the samples were subjected to western blot with indicated antibodies.

### Cell transfection and lentivirus package

For RNA interference by lentiviral vectors, *CDK9* shRNA constructs and a negative control construct created in the same vector system (pLKO.1) were purchased from Corues Biotechnology. Cells were co-transfected with shRNA constructs (10 μg) together with Lentiviral Mix (10 μL) and HG Transgene TM Reagent (60 μL), according to the manufacturer’s instruction of Lentiviral Packaging Kit (YEASEN). Viral supernatant was harvested from the culture medium 48 h after transfection. And then infected target cells with virus supernatant and selected 2 μg/mL puromycin for screening.

### Quantitative real-time RT-qPCR

RT-qPCR was performed according to the manufacturer’s instructions [29]. The primer sequences were as follows:

Human *CDK9*: forward: 5'- AAAGTCTGCCAGCTTCAGGA -3'.
  reverse: 5'- ACTTCTGCGAGCATGACCTT -3'.
Human *GAPDH*: forward: 5'-TGATGACATCAAGAAGGTGGTGAA -3'.
  reverse: 5'- TCCTTGGAGGCCATGTGGGCCAT -3'.

### Immunofluorescence analysis

Immunofluorescence assays were performed as previously reported [30]. Images were observed with a confocal microscope (FV1000; Olympus, Tokyo, Japan).

#### TUNEL assay

Apoptosis was determined using a One Step TUNEL apoptosis assay kit (Beyotime, Jiangsu, China). A double-staining technique was used; TUNEL staining (green fluorescence) was used to quantitate apoptotic cell nuclei and DAPI staining (blue fluorescence) was used to quantitate the total cell nuclei, as previously described [31]. The stained samples were observed under a confocal microscope (FV1000; Olympus, Tokyo, Japan). Three visual fields were selected randomly for each specimen.

#### Animal models

The female *NOD/SCID* mice (5 weeks old, weighing 16–20 g) (Cavens Changzhou Laboratory Animal Co., Ltd, Jiangsu, China) were sublethally irradiated (1.8 Gy) and were engrafted with KU-812 cells (6 × 10^6^) via subcutaneously injection within 24 h following the radiation treatment. After 10 days, the mice were divided randomly into three groups (n = 6 per group): a control group (0.9% normal saline), a wogonin-treated group (80 mg/kg) and an Imatinib-treated group (200 mg/kg). The animals in wogonin groups were intraperitoneal injection (i.p) of wogonin (80 mg/kg, every day for 2 weeks). The animals in Imatinib-treated group were received administrated orally of Imatinib (200 mg/kg, every other day for 2 weeks). The tumor volume of mice was measured daily with an electronic vernier caliper. Finally, the animals were sacrificed, and the tumors were prepared for western blots and immunofluorescence assays, the spleens were prepared for flow cytometric analysis after the cells labeled with human CD45-PE (huCD45-PE) and the survival status are counted. The staining of huCD45-PE is used to determine the infiltration of KU-812 cells.

Animals were maintained in an air-conditioned and pathogen-free environment (23 ± 2 °C, 55 ± 5% humidity) under controlled lighting (12 h light/day) and supplied with standard laboratory food and water throughout the experimental period. The animal study was carried out according to the regulations of the China Food and Drug Administration (CFDA) on Animal Care.

#### FACS analysis of cells extracted from spleens

Cells extracted from spleens were performed as previously reported [32]. FACS Calibur flow cytometry was used to detect huCD45 positive cells. In this model, human CD45 positive cells were defined as human leukemia cells.

#### Statistical analysis

All results are expressed as the mean ± SD from at least three independent experiments performed in a parallel manner. Statistical analyses of multiple group comparisons were performed by one-way analysis of variance followed by the Bonferroni *post-hoc* test. Comparisons between two groups were analyzed using two-tailed Student’s *t* tests. The significance of differences is indicated as *p < 0.05, **p < 0.01 and ***p < 0.001.

## Result

### Wogonin induced erythroid differentiation in chronic myeloid leukemia cell lines and primary CML cells

**Fig. 1:**
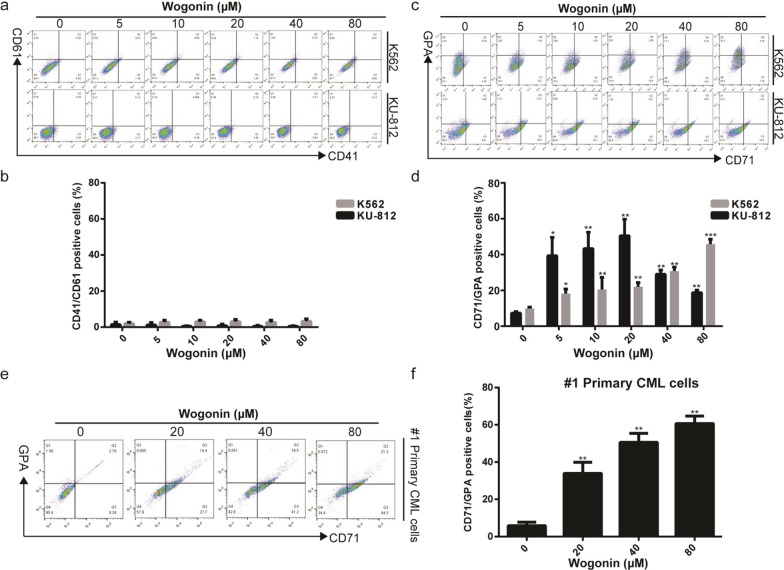
Wogonin induced erythroid differentiation in chronic myeloid leukemia cell lines and primary CML cells. **a** K562 and KU-812 cells were treated with wogonin (0, 5, 10, 20, 40 and 80 μM) for 120 h. The expression of CD41 and CD61 was detected by flow cytometry analyses. **b** Quantification of the expression of CD41/CD61. Columns represent the mean from three parallel experiments (mean ± SD), compared with control group. **c** K562 and KU-812 cells were treated with wogonin (0, 5, 10, 20, 40 and 80 μM) for 120 h. The expression of CD71 and GPA was detected by flow cytometry analyses. **d** Quantification of the expression of CD41/CD61. Columns represent the mean from three parallel experiments (mean ± SD). *p < 0.05, **p < 0.01, ***p < 0.001, compared with control group. **e** #1 Primary CML cells were treated with wogonin (0, 20, 40 and 80 μM) for 120 h. The expression of CD71 and GPA was detected by flow cytometry analyses. **f** Quantification of the expression of CD41/CD61. Columns represent the mean from three parallel experiments (mean ± SD). *p < 0.05, **p < 0.01, compared with control group

In order to investigate anti-CML effects of wogonin, we first detect differentiation on K562, KU-812 and primary CML cells. K562 and KU-812 cells were treated with indicated concentrations of wogonin (0, 5, 10, 20, 40, 80 μM) for 120 h. We examined the expression of CD41 and CD61, two megakaryocytic differentiation specific markers. However, the numbers of CD41 and CD61 positive cells did not have significant increased after wogonin treatment in K562 and KU-812 cells (Fig. 1a, b). We then detected the expression of CD71 and GPA, two erythroid differentiation specific markers. As shown in Fig. 1c, d, treatment with wogonin increased the proportion of CD71- and GPA-positive cells in K562 cells and KU-812 cells. In #1 primary CML cells, wogonin induced erythroid differentiation in a concentration-dependent manner (Fig. 1e, f). We concluded that wogonin induced erythroid differentiation but not megakaryocytic differentiation in K562, KU-812 and primary CML cells as judged by flow cytometry analyses.

#### P-TEFb was involved in wogonin-induced erythroid differentiation and regulated the protein interaction of GATA-1, RUNX1 and FOG-1

P-TEFb is a general transcription factor that regulates transcription elongation through phosphorylation of the C-terminal tail domain (CTD) of RNA polymerase II (RNAP II), it is a heterodimer kinase composed of CDK9 and its regulated subunit Cyclin T1 [33]. For verifying whether erythroid differentiation associated with CDK9 on chronic myeloid leukemia cell lines and primary CML cells, we first constructed lentiviral recombinants expressing shRNA targeting *CDK9* gene on K562 cells and evaluated knock down efficiency by using RT-qPCR (Additional file 1: Fig. S1a). Then we also tested the expression of RNA Pol II, the phosphorylation level of RNA polymerase II (RNA Pol II Ser2 and Ser5) by western blot. Results showed that the expression of phosphorylation forms of RNA polymerase II Ser2 and Ser5 were both downregulated compared to negative control group, it was indicated that P-TEFb kinase activity was inhibited when *CDK9* was knocked down (Additional file 1: Fig. S1b). To further confirm the involvement of *CDK9* on erythroid differentiation in CML cells, we detected the expression of surface antigens (CD71 and GPA) by flow cytometry analyses. Results showed that the ratio of erythroid differentiation was increased to 56.85 ± 3.19% in sh*CDK9* group compared to negative control group (Additional file 1: Fig. S1c, d).

**Fig. 2:**
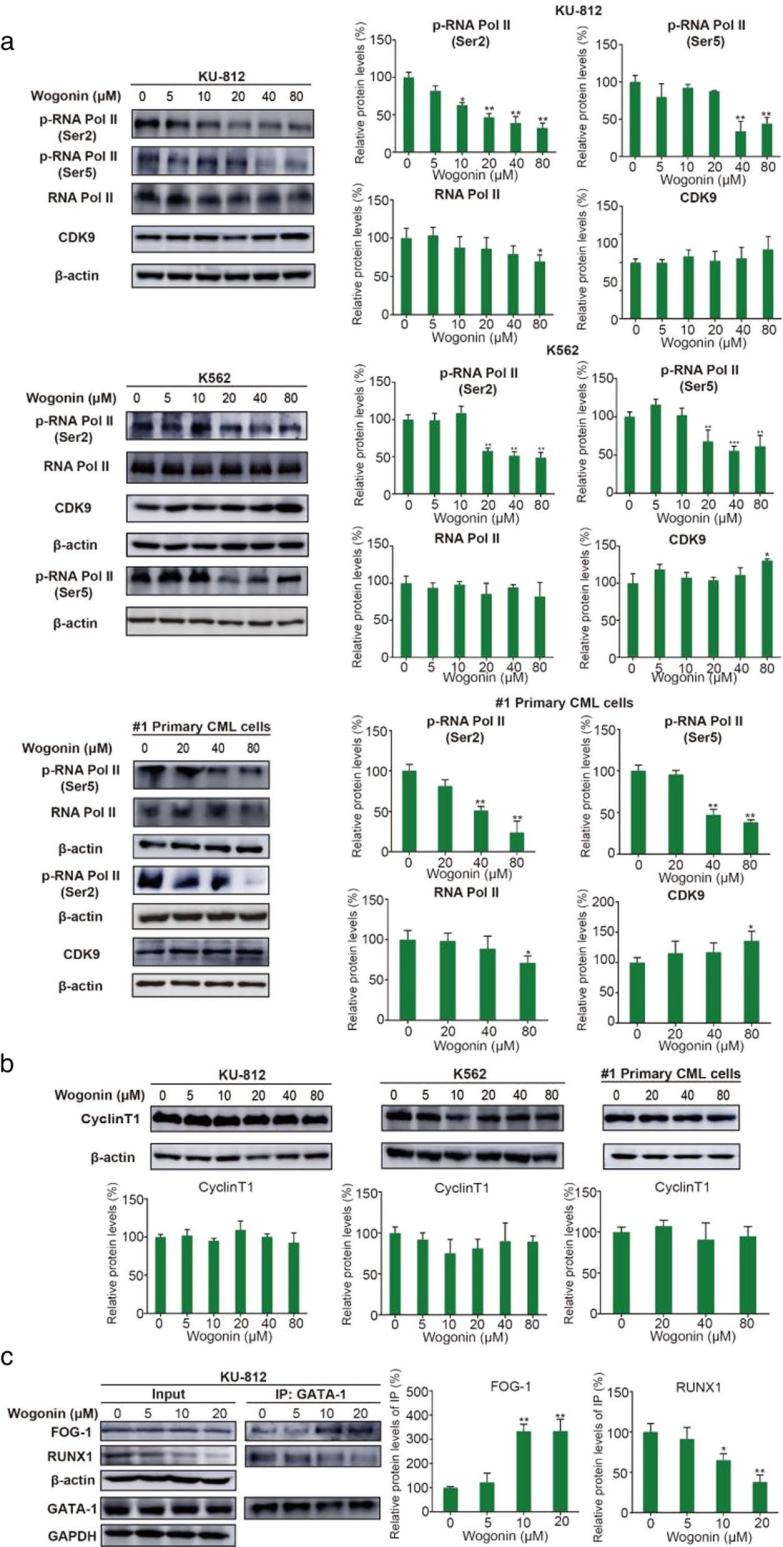
P-TEFb was involved in wogonin-induced erythroid differentiation and regulated the protein interaction of GATA-1, RUNX1 and FOG-1. **a** After treatment with wogonin (0, 5, 10, 20, 40 and 80 μM) for 48 h in K562 and KU-812 cells, and in #1 primary CML cells wogonin (0, 20, 40, 80 μM) was treated for 48 h. Protein expression levels of p-RNA Pol II (Ser2), p-RNA Pol II (Ser5), RNA Pol II and CDK9 were analyzed by western blot. β-actin was used as a loading control. The results are representative of three independent experiments(mean ± SD). *p < 0.05, **p < 0.01, ***p < 0.001, compared with control group. **b** After treatment with wogonin (0, 5, 10, 20, 40 and 80 μM) for 48 h in K562 and KU-812 cells, and in #1 primary CML cells wogonin (0, 20, 40, 80 μM) was treated for 48 h. Protein expression levels of Cyclin T1 were analyzed by western blot. β-actin was used as a loading control. The results are representative of three independent experiments. (mean ± SD). *p < 0.05, **p < 0.01, compared with control group. **c** KU-812 cells were treated with wogonin (0, 5, 10 and 20 μM) for 72 h. Cells were immunoprecipitated with anti-GATA-1 antibody, followed by western blot analysis with anti-FOG-1 and anti-RUNX1 antibodies. GAPDH and β-actin were used as a loading control respectively. The results are representative of three independent experiments (mean ± SD). *p < 0.05, **p < 0.01, compared with control group

In previous studies we have already known wogonin is a CDK9 inhibitor, which directly binds to the ATP-binding pocket of CDK9 [34]. In order to investigate P-TEFb kinase activity involving in wogonin-induced erythroid differentiation, we examined the expression of the CTD of RNA Pol II in these cells. Western blot showed that the protein levels of RNA Pol II had slightly decreased and its phosphorylated forms (Ser2 and Ser5) were significantly decreased after treatment with various concentrations of wogonin (0, 5, 10, 20, 40, 80 μM) for 48 h in KU-812 cells (Fig. 2a). In K562 cells, the protein levels of RNA Pol II did not significantly change but its phosphorylated form (Ser2 and Ser5) were significantly decreased after treatment with various concentrations of wogonin for 48 h (Fig. 2a). The expression of CDK9 slightly increased after treatment of 80 μM wogonin on K562 but in KU-812 cells the expression of CDK9 had no statistically significant difference (Fig. 2a). Similarly, in #1 primary CML cells wogonin reduced the expression of RNA Pol II and its phosphorylation form (RNA Pol II Ser2 and Ser5), the level of CDK9 had slight increased by treatment with 80 μM wogonin (Fig. 2a). Moreover, the expression of Cyclin T1 had no significant change in K562, KU-812 and #1 primary CML cells (Fig. 2b). It is indicated that wogonin inhibited the kinase activity of P-TEFb by reducing the expression of the phosphorylation forms of RNA Pol II, which is a substrate protein of P-TEFb.

Some studies have shown that P-TEFb mediates the binding of GATA-1 and RUNX1, which promotes megakaryocytic differentiation and inhibits erythrocyte differentiation [35]. FOG-1 (Friend of GATA-1) specifically binds to the N-terminal zinc finger structure of GATA-1, the protein interaction of GATA-1 and FOG-1 plays an essential role in erythrocyte differentiation [36]. Therefore, for further study the mechanism of wogonin-induced erythroid differentiation, co-IP was used to investigate the protein interaction of GATA-1- FOG-1 and GATA-1-RUNX1 in KU-812 cells. As anticipated, wogonin increased the binding capacity of GATA-1 and FOG-1, however wogonin dramatically decreased the binding capacity of GATA-1 and RUNX1 (Fig. 2c).

#### Wogonin induced apoptosis in KU-812 cells through downregulating *MCL1*

**Fig. 3:**
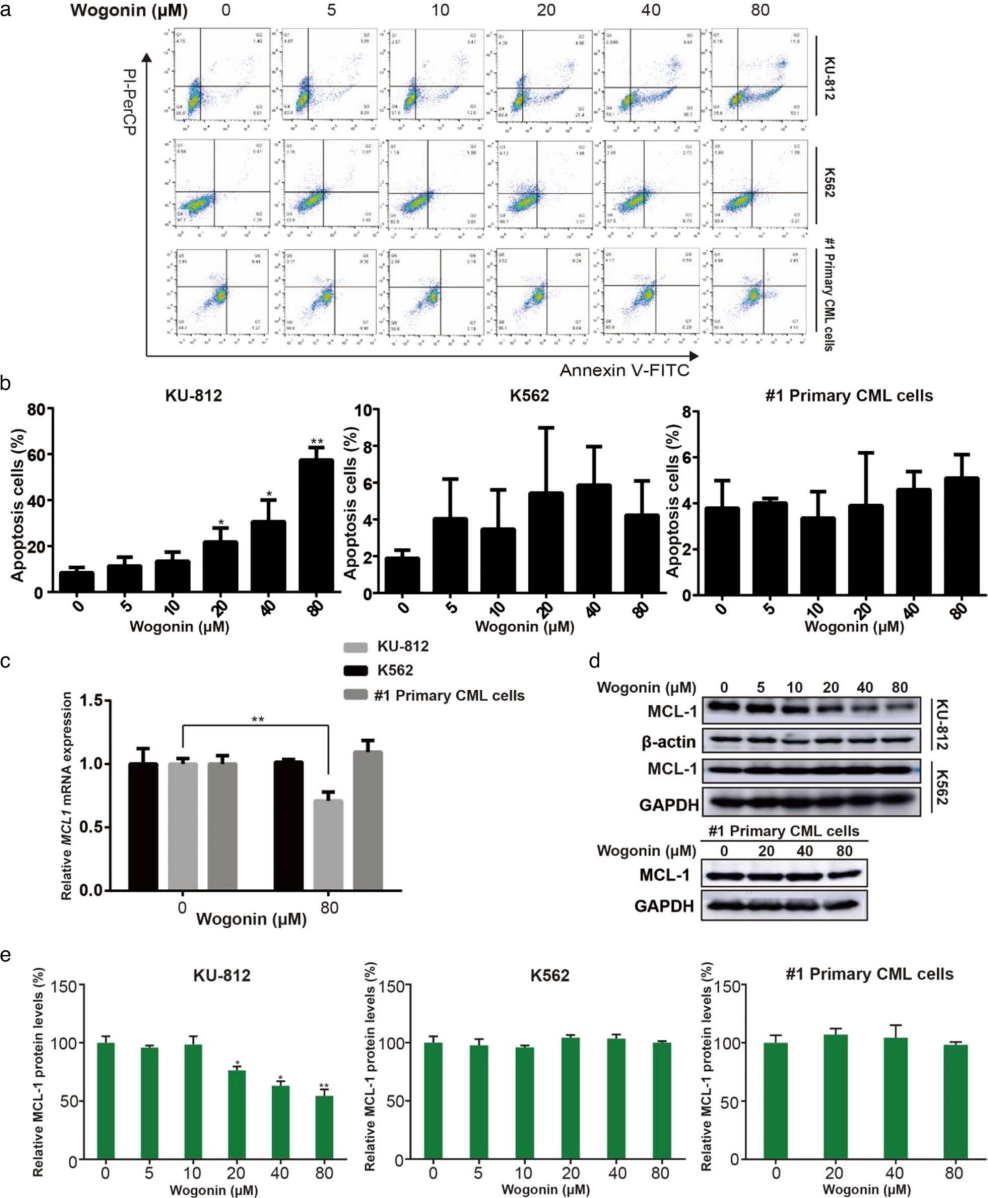
Wogonin induced apoptosis in KU-812 cells *through downregulating MCL1*. **a** KU-812, K562 and #1 primary CML cells were treated with Wogonin (0, 5, 10, 20, 40 and 80 μM) for 48 h. Flow cytometric analysis of Annexin V/PI stained cells. Data represent the mean ± SD of three independent experiments. **b** Quantification of apoptosis cells. Columns represent the mean from three parallel experiments (mean ± SD). *p < 0.05, **p < 0.01, compared with control group. **c** The mRNA level of *MCL1* in KU-812, K562 cells and #1 primary CML cells after cells were treated with wogonin (0 and 80 μM) for 48 h. Columns represent the mean from three parallel experiments (mean ± SD). **p < 0.01, compared with control group. **d** The protein expression level of MCL-1 in KU-812, K562 cells and #1 primary CML cells after cells were treated with various concentrations of wogonin for 48 h. β-actin and GAPDH was used as a loading control respectively. The results are representative of three independent experiments. **e** Quantification of the protein expression level of MCL-1 in KU-812, K562 cells and #1 primary CML cells. Columns represent the mean from three parallel experiments (mean ± SD). *p < 0.05, **p < 0.01, compared with control group

To further investigate the effect of apoptosis in CML cells, Annexin V/PI double-staining assay was conducted in K562, KU-812 cells and #1 primary CML cells treated by various concentrations of wogonin (0, 5, 10, 20, 40, 80 μM) for 48 h. Results showed that high concentrations of wogonin (40, 80 μM) induced more obvious apoptosis in KU-812 cells, but did not in K562 and #1 primary CML cells (Fig. 3a, b). Moreover, after treatment of wogonin (80 μM) for 48 h, KU-812 cells were dyed for TUNEL-FITC, compared with control group, wogonin treatment group was TUNEL-positive and the cells showed more green fluorescence, it was indicated that wogonin could induce apoptosis in KU-812 cells (Additional file 3: Fig. S3). Furthermore, as a downstream target gene of RNA Pol II, *MCL1* is closely related the survival and maintenance of tumor cells [33]. In order to examine the mRNA level of *MCL1*, we first used RT-PCR to detect the expression of *MCL1* after treatment of wogonin. Results showed that 80 μM wogonin inhibited transcriptional activity of *MCL1* in KU-812 cells, but in K562 and primary CML cells no significant change was shown (Fig. 3c). Moreover, results of western blot showed that wogonin decreased the protein expression of MCL-1 in KU-812 cells in a concentration-dependent manner (Fig. 3d,e). Similar with the level of mRNA, wogonin had no effect on the protein expression of MCL-1 in K562 and #1 prima ry CML cells (Fig. 3d, e). Therefore, the downregulation of *MCL1* would be the cause of wogonin-induced apoptosis in KU-812 cells.

#### Wogonin exhibited anti-CML effects and exerted lower toxicity in vivo

**Fig. 4:**
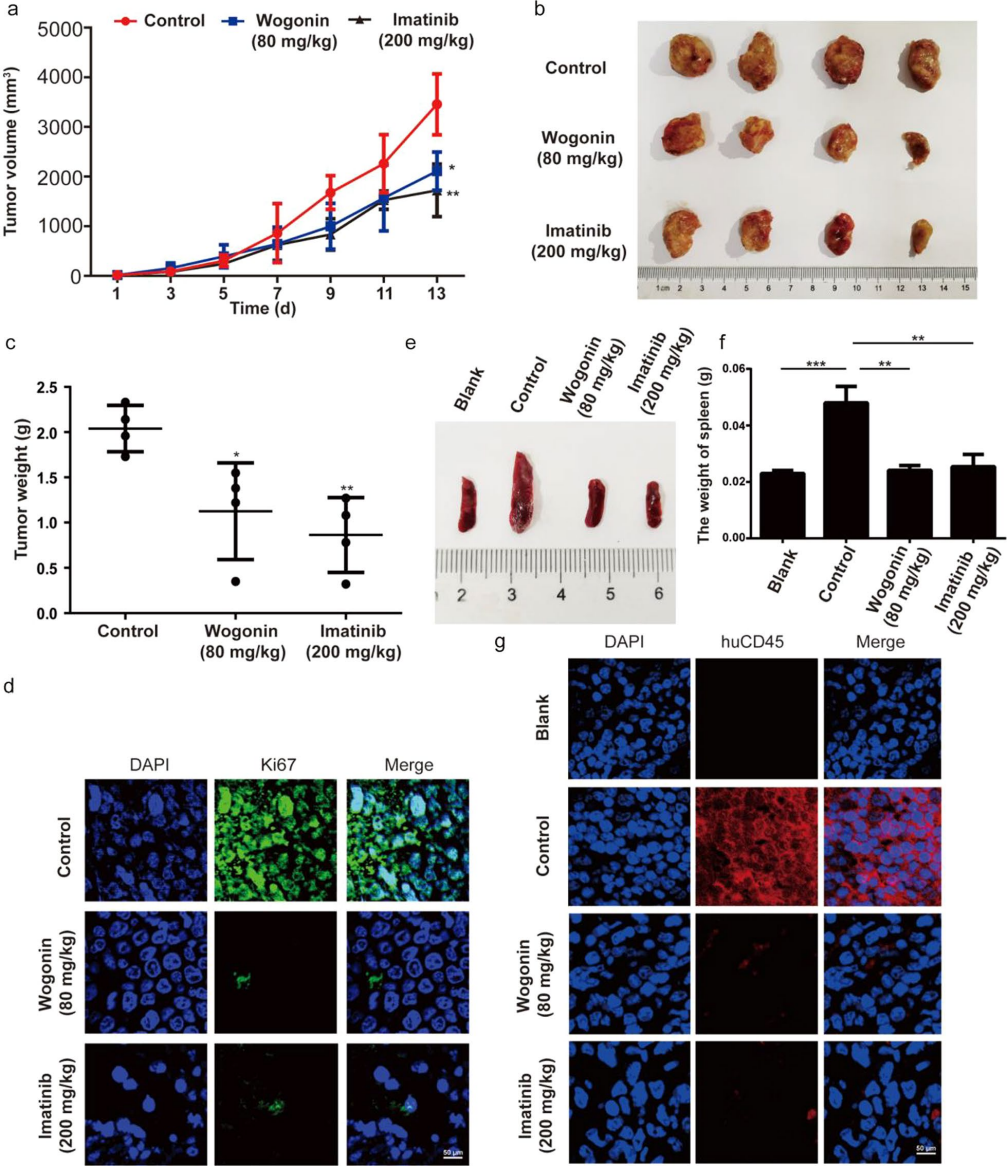
Wogonin exhibits anti-tumor effects and exerted lower toxicity in vivo*.* In total, KU-812 cells (6 × 10^6^ cells/mouse) were subcutaneously inoculated into *NOD/SCID* mice. The mice were randomized into 6 groups (6 mice per group), and treated with wogonin (80 mg/kg), Imatinib (200 mg/kg) and 0.9% normal saline for 2 weeks. **a** The tumor volume was measured and calculated every other day. Data represent mean ± SD of three independent experiments. *p < 0.05, **p < 0.01, compared with control group. **b** The resulting tumors excised from the animals after treatment. **c** The tumor weights in three groups were compared. Quantification of the data shown. Data represent mean ± SD of three independent experiments. *p < 0.05, **p < 0.01, compared with control group. **d** Effect of wogonin on expression of Ki67 in KU-812 xenografts (Original magnification × 600). **e**–**f** Wogonin inhibits the splenomegaly of KU-812-bearing *NOD/SCID* mice. The typical photos of the spleen. Effect of wogonin on the weight of spleen in KU-812 cells-bearing *NOD/SCID* mice. Each data point represents the mean ± SD of four animals for each group. **p < 0.01, ***p < 0.001, compared with control group. **g** Effect of wogonin on expression of huCD45^+^ in spleen of KU-812-bearing *NOD/SCID* mice. Spleen samples from three mice of each group (Original magnification × 600)

In order to confirm wogonin-induced anti-CML effects in vivo, we constructed *NOD/SCID* mouse models engrafted with KU-812 cells via subcutaneous transplantation. The animals in control and wogonin-treated groups were intraperitoneal injection with solvent or wogonin (80 mg/kg, every day), and the animals in Imatinib-treated group were administrated orally (200 mg/kg, every other day). After administration for two weeks, we killed them and the graft tumors were taken out and weighed. As was indicated by tumor pattern, wogonin exhibited an observable tumor inhibition effect in KU-812 xenografts compared with the control group (Fig. 4a, b, c). Compared with Imatinib-treated group, the effect of wogonin-treated group was almost the same (Fig. 4a, b, c). The expression of Ki67 also indicated that both of wogonin and Imatinib possessed strong anti-proliferation activity (Fig. 4d). Moreover, the spleens of each group of mice were dissected and observed. Mice inoculated with KU-812 cells had significantly enlarged spleens, and splenomegaly was effectively alleviated in the treatment groups with wogonin and Imatinib (Fig. 4e, f). We also collected spleens randomly for biopsy which were selected from each group. Results of immunofluorescence showed that compared with mice in the blank group, the numbers of huCD45^+^ cells in spleens of mice inoculated with KU-812 cells were significantly increased in the control group, while the numbers of huCD45^+^ cells in wogonin and Imatinib-treated group were significantly decreased (Fig. 4g). For investigating whether wogonin had obvious toxicity on mice, we therefore assessed the effect of wogonin on animals’ weight and main organs. As indicated in Additional file 2: Fig. S2a, there was a weight drop of mice in the Imatinib-treated group, while no significant weight loss was observed in wogonin group. According to hematoxylin and eosin staining of three organs from each mouse, we found that wogonin exerted lower toxicity whereas Imatinib-treated group displayed injuries of varying intensity (Additional file 2: Fig. S2b).

#### Wogonin modulated P-TEFb kinase activity in vivo

**Fig. 5:**
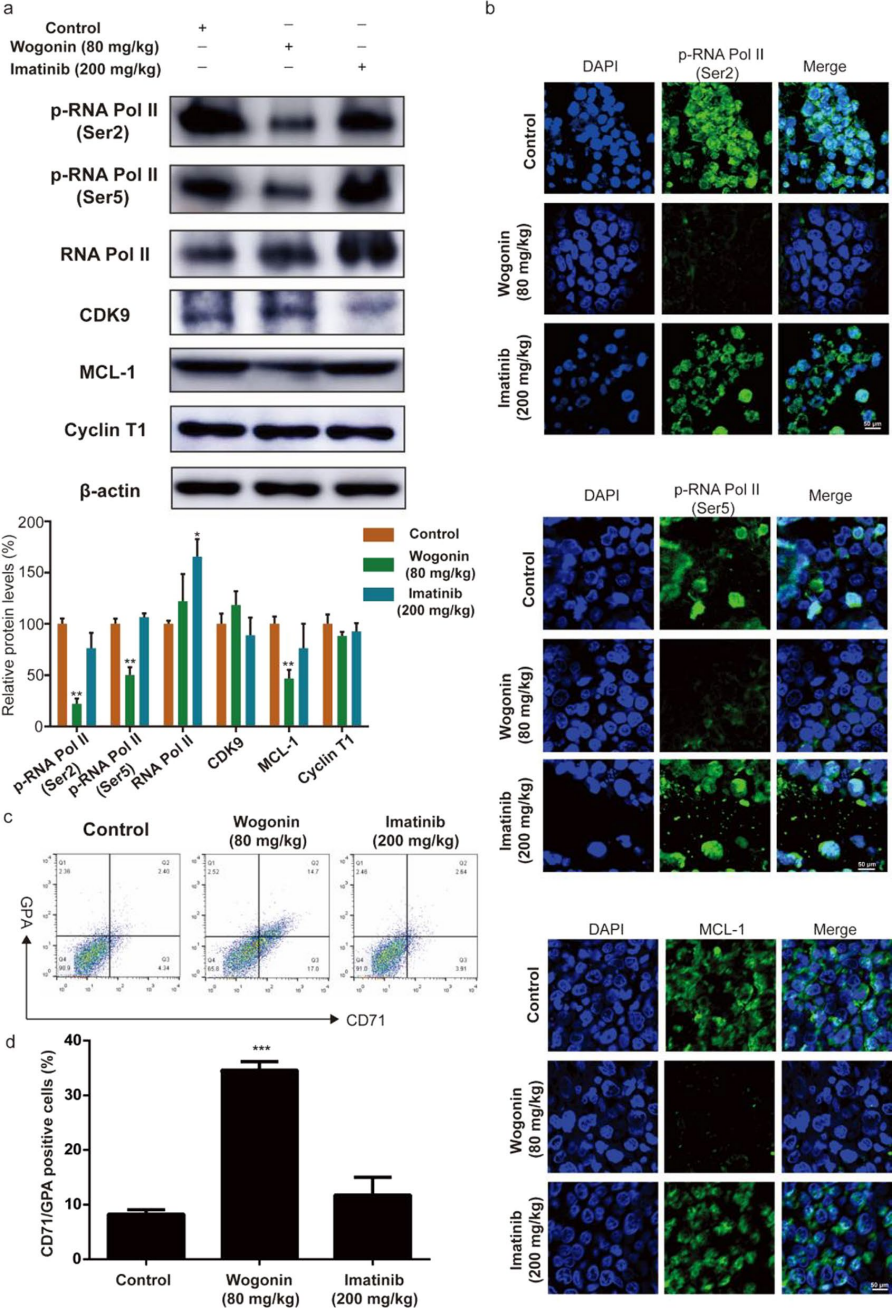
Wogonin modulated P-TEFb kinase activity in vivo*.* **a** The protein expression of p-RNA Pol II (Ser2), p-RNA Pol II (Ser5), CyclinT1, CDK9 and MCL-1 in KU-812 xenografts. β-actin was used as a loading control. The results are representative of three independent experiments (mean ± SD). *p < 0.05, **p < 0.01, compared with control group. **b** Immunofluorescence and stained with p-RNA Pol II (Ser2) (green fluorescence), p-RNA Pol II (Ser5) (green fluorescence) and MCL-1 (green fluorescence) respectively, as well as DAPI (blue fluorescence) in tumor of KU-812-bearing *NOD/SCID* mice. They were detected by confocal microscopy (FV1000; Olympus) with FV10-ASW2.1 acquisition software (Olympus) at room temperature (Original magnification × 600). **c** The expression of CD71 and GPA was detected in tumor of KU-812-bearing *NOD/SCID* mice by flow cytometry analyses. **d** Quantification of the expression of CD71/GPA. Data represent mean ± SD of three independent experiments. ***p < 0.001, compared with control group

In order to verify the mechanism of wogonin-induced anti-CML activity in vivo, we detected P-TEFb kinase associated proteins in KU-812 transplanted tumors by western blot. Results showed that the expression of the phosphorylation forms of RNA polymerase II (p-RNA Pol II Ser2 and Ser5) and MCL-1 were decreased in wogonin-treated group compared to control group, which was consistent with the results in vitro (Fig. 5a). The expression of CDK9 and cyclin T1 did not have significant changes in wogonin-treated group (Fig. 5a). Moreover, immunofluorescence experiments also confirmed the decreased effect of wogonin on the expression of p-RNA Pol II (Ser2), p-RNA Pol II (Ser5) and MCL-1 in KU-812 cells xenografts (Fig. 5b).

For demonstrating erythroid differentiation induced by wogonin in vivo, we detected surface antigens CD71 and GPA in cells extracted from tumor issues of KU-812 cells-bearing *NOD/SCID* mice. Results showed that the GPA/CD71 positive ratio in wogonin-treated group increased from 9.4 ± 0.36% to 34.60 ± 1.57% compared with control group, but in Imatinib-treated group no effect was observed (Fig. 5c, d). All these results indicated that wogonin could reduce the growth of CML cells by modulating P-TEFb kinase in vivo.

## Discussion

Although classic TKIs targeting BCR-ABL1 in CML cells makes patient’s condition acquired greatly improved, but is not curative and frequently limited by intolerance or resistance [37]. Differentiation therapy remains a classical and unsurpassable therapeutic schedule in hematologic malignancies [38]. All-*trans* retinoic acid in treating acute promyelocytic leukemia (APL) results in cure rates of over 80% [39]. In this study, we found wogonin, a traditional flavonoid, induced differentiation and apoptosis in CML cells lines and primary CML cells mediated by P-TEFb. Moreover, wogonin inhibited the growth of KU-812 xenografts mediated by P-TEFb was also verified in vivo.

P-TEFb complex is a special kinase that plays a key role in transcription elongation. RNA pol II is the main functional target of the complex. GATA-1, which is a key transcription factor that interacts with a variety of proteins including FOG-1 and RUNX1 to regulate erythroid and megakaryocyte differentiation respectively [35, 40]. However, FOG-1 negatively regulates RUNX1-GATA-1 cooperation, which attains the switch between erythroid and megakaryocyte differentiation [35]. The interaction of GATA-1 with RUNX1 is highly dependent on the P-TEFb kinase complex [22, 35]. It has been reported that wogonin induced erythroid differentiation in CML cells [41]. CDK9 is a core component of P-TEFb by binding with cyclinT1 to form an active P-TEFb kinase complex. Previous studies have shown that wogonin has a good inhibitory effect on CDK9 kinase [34]. To this end, we suspected that wogonin induced erythroid differentiation in CML cells involving modulation on P-TEFb.

GATA-1 is the key node of megakaryocyte / erythroid differentiation, and the formation of GATA-1/RUNX1 complex, which is necessary for megakaryocyte differentiation, depends on the activity of P-TEFb. However, whether inhibition of P-TEFb activity can promote erythroid differentiation remains unknown. Thus, we first used *CDK9*-shRNA to knock down the expression of CDK9 and results showed that erythroid differentiation was increased in *CDK9*-shRNA group compared to scramble group. Moreover, the expression of the downstream target of CDK9, the phosphorylated forms (Ser2 and Ser5) of RNA pol II were also decreased in *CDK9*-shRNA group. It was indicated that P-TEFb kinase activity was associated with erythroid differentiation in CML cells. Then we found wogonin decreased the phosphorylated forms (Ser2 and Ser5) of RNA pol II in a concentration-dependent manner but did not change the expression of cyclin T1 in CML cell lines and primary CML cells. It was indicated that wogonin inhibited P-TEFb activity in CML cells. Moreover, co-immunoprecipitation assay showed that wogonin decreased the interaction of GATA-1 and RUNX1 but increased the interaction of GATA-1 and FOG-1. The results demonstrated that wogonin promoted erythroid differentiation but repressed megakaryocyte differentiation, which could attribute to the inhibition of P-TEFb activity. We also verified the inhibition of P-TEFb activity in vivo, meanwhile, erythroid differentiation was also increased by wogonin in xenograft of KU-812 cells.

The transcription of protein with short half-lives like MCL-1 is prolonged via P-TEFb, which phosphorylates the carboxy terminal of RNA polymerase II [33, 42]. CDK9 is a critical component of P-TEFb. In this context, inhibition of CDK9 by wogonin blocked the activity of RNA pol II and induced apoptosis in KU-812 cells. As the downstream of RNA pol II, the transcriptional level of *MCL-1* was decreased in KU-812 cells. Consistent with that the protein level of MCL-1 also decreased in KU-812 cells. Moreover, we verified the decreased expression of MCL-1 in in KU-812 cells xenografts. It’s worth mention that the protein expression of MCL-1 was not significant decreased in vivo of Imatinib treated group (Fig. 5a, b). In general, some studies showed MCL-1 is a BCR-ABL dependent target in CML cells and TKIs could decreased the expression of MCL-1 [43]. However, heterogeneity of tumors in vivo study may cause abnormal expression of MCL-1 in Imatinib treated group. As a matter of fact, we found some studies showed MCL-1 expression elevated after Imatinib compared with control group in CML patients, which was linked to the drug resistance and individual difference [44].

In K562 and #1 primary CML cells, wogonin could not induce apoptosis and decrease the expression of MCL-1. We observed that in K562 cells wogonin-induced erythroid differentiation was concentration-dependent but in KU-812 cells, the degree of erythroid differentiation was gradually increased by the concentration of 0, 5, 10, 20 μM but decreased when the concentration was 40 and 80 μM. Although wogonin inhibited the kinase activity of P-TEFb in CML cell lines and primary CML cells, MCL-1 is one of downstream target genes of RNA Pol II. As a matter of fact, a number of growth factors, cytokines and cytotoxic stimuli (e.g. drugs, radiation) regulate MCL-1 transcription through cell-type dependent effects on signal transduction pathways such as the PI3K/Akt, JAK/STAT, p38/MAPK, and MEK/ERK pathways, with both antiapoptotic and proapoptotic stimuli involved [45, 46].Thus not only RNA Pol II but also different types of signaling pathways may be involved in the regulation of MCL-1 expression, which depends on the cell types. In our study, we speculated that wogonin-induced different effects on MCL-1 may be regulated by different signaling pathways, which is not limited to RNA Pol II. Some studies showed that inhibiting apoptosis by ZVAD-fmk allows the surviving cells to differentiate towards the erythroid lineage, to some extent, BCR-ABL1-mediated apoptosis and erythroid differentiation are two independent processes which can be fully distinguished notably regarding their sensitivity to caspase and p38 MAP kinase inhibition. [47, 48]. It has been reported that overexpression of MCL1 may inhibit apoptosis and subsequent inhibition of apoptosis may support cellular cell differentiation of CML cells [49]. An investigation showed that one murine cell line, ELM-I-1, required both activin A and EPO to achieve erythroid differentiation, however, apoptosis was induced by treatment with activin A alone. On the contrary, in another murine cell line, F5-5, differentiation was sufficiently induced by activin A alone to achieve an erythroid lineage. One possible explanation for these differences in activin A reactivity in these two cell lines was the state of the EPO receptor [50]. As observed in the case of wogonin in three types of cells, the differences among these cells like the state of the EPO receptor or cell sensitivity to wogonin may cause the different response degree in different cells, resulting in the transition of cell death and differentiation. The specific mechanism of the transformation of these two effects remains to be further explored.

**Fig. 6:**
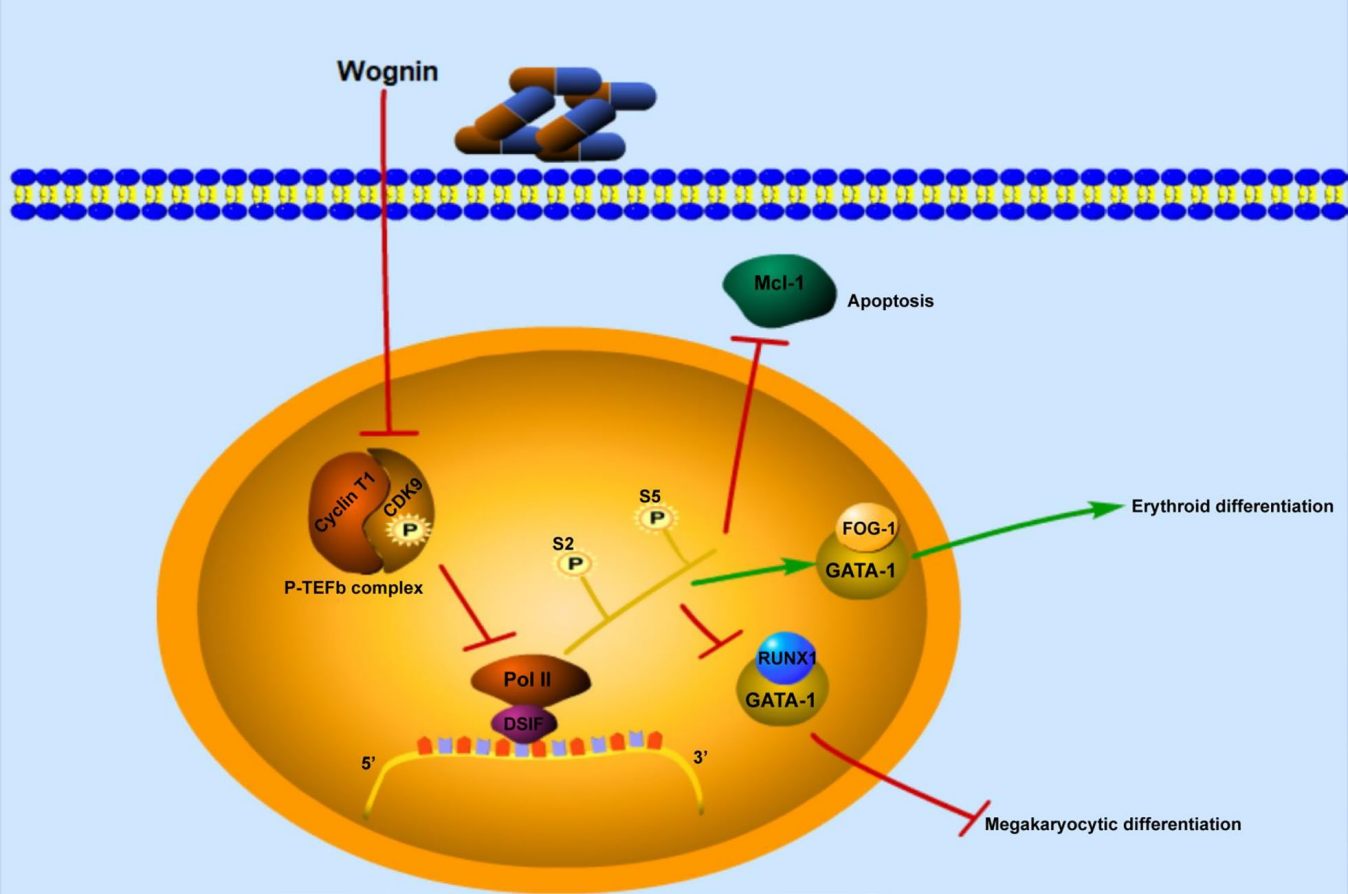
Graphical abstract on the mechanisms of pharmacologic targeting of the P-TEFb complex induced by wogonin on CML cells

Taken together, our results showed the involvement of P-TEFb in wogonin-induced apoptosis and erythroid differentiation in CML cells. Furthermore, we demonstrated that wogonin induced-erythroid differentiation was associated with the increasing interaction of GATA-1 and FOG-1, which were regulated by P-TEFb. This research indicated that wogonin showed good inhibitory effect on CML cells through this unique mechanism, suggesting the potential application of wogonin in developing into a novel agent for the treatment of CML (Fig. 6).

## Conclusions

Our research elucidated the regulatory mechanism of P-TEFb and GATA-1 signal crosslinking in the erythroid differentiation of CML cells induced by wogonin. Moreover, we demonstrated that wogonin induced erythroid differentiation of CML cells by inhibiting P-TEFb kinase activity, reducing the interaction between GATA-1 and RUNX-1, and promoting the binding of GATA-1 to FOG-1. This study highlighted a role of P-TEFb in wogonin-treated CML cells and provided a potential mechanism for the application of wogonin.

## Supplementary Information

**Additional file 1**. **Figure S1**. CDK9 shRNA K562 cells enhanced the rates of erythroid differentiation. (a) K562 cells were transfected with NC shRNA or CDK9 shRNA. The mRNA level of CDK9 was detected by RT-PCR. Data represent mean ± SD of three independent experiments. ***p < 0.001, compared with control group. (b) In control, NC and shCDK9 groups, cell lysates were analysed for p-RNA Pol II (Ser2), p-RNA Pol II (Ser5), RNA Pol II and CDK9 expression by western Blot. Data represent mean ± SD of three independent experiments. *p < 0.05, **p < 0.01, compared with NC group. (c) In control, NC and shCDK9 groups, the expression of CD71 and GPA was detected by flow cytometry analyses. Data represent the mean ± SD of three independent experiments. (d) Quantification of the expression of CD71/GPA. Data represent mean ± SD of three independent experiments. ***p < 0.001, compared with NC group.

**Additional file 2**. **Figure S2**. Toxicological assessment. (a) NOD/SCID mice weights were recorded every 2 days. (b) H&E stained main organs of mice from treated and control group to evaluate the toxicity of wogonin.

**Additional file 3**. **Figure S3**. Apoptosis detection of KU-812 cells by TUNEL (a) KU-812 cells were treated with wogonin (0, 80 μM) for 48 h. Cell apoptosis was measured by TUNEL staining used a confocal microscope. Three visual fields were selected randomly for each specimen.

## Data Availability

The datasets supporting the conclusions of this article are included within the article and its additional files.

## Abbreviations

CML: Chronic myeloid leukemia
P-TEFb: The positive transcription elongation factor b
CDK9: Cyclin-dependent kinase 9
CDKs: Cyclin-dependent kinases
RNA Pol II: RNA Polymerase II
CTD: C-Terminal Domain
APL: Acute promyelocytic leukemia
RA: Retinoic acid
CMP: Common myeloid progenitor
MEP: Megakaryocyte erythrocyte progenitor
GRA: Granulocyte macrophage progenitor
GATA-1: GATA binding protein 1
FOG-1: Friend of GATA-1
RUNX1: Runt-related transcription factor 1
RT-qPCR: Real-time quantitative polymerase chain reaction
TUNEL: TdT-mediated dUTP nick-end labeling
HSC: Hematopoietic stem cell
