# Supplementary figures and images for: Pharmacologic targeting of the P-TEFb complex as a therapeutic strategy for chronic myeloid leukemia
Source: Cell Commun Signal. 2021 Aug 9;19:83. doi: 10.1186/s12964-021-00764-5 (PMC8351106; doi:10.1186/s12964-021-00764-5)

**Figure S1**

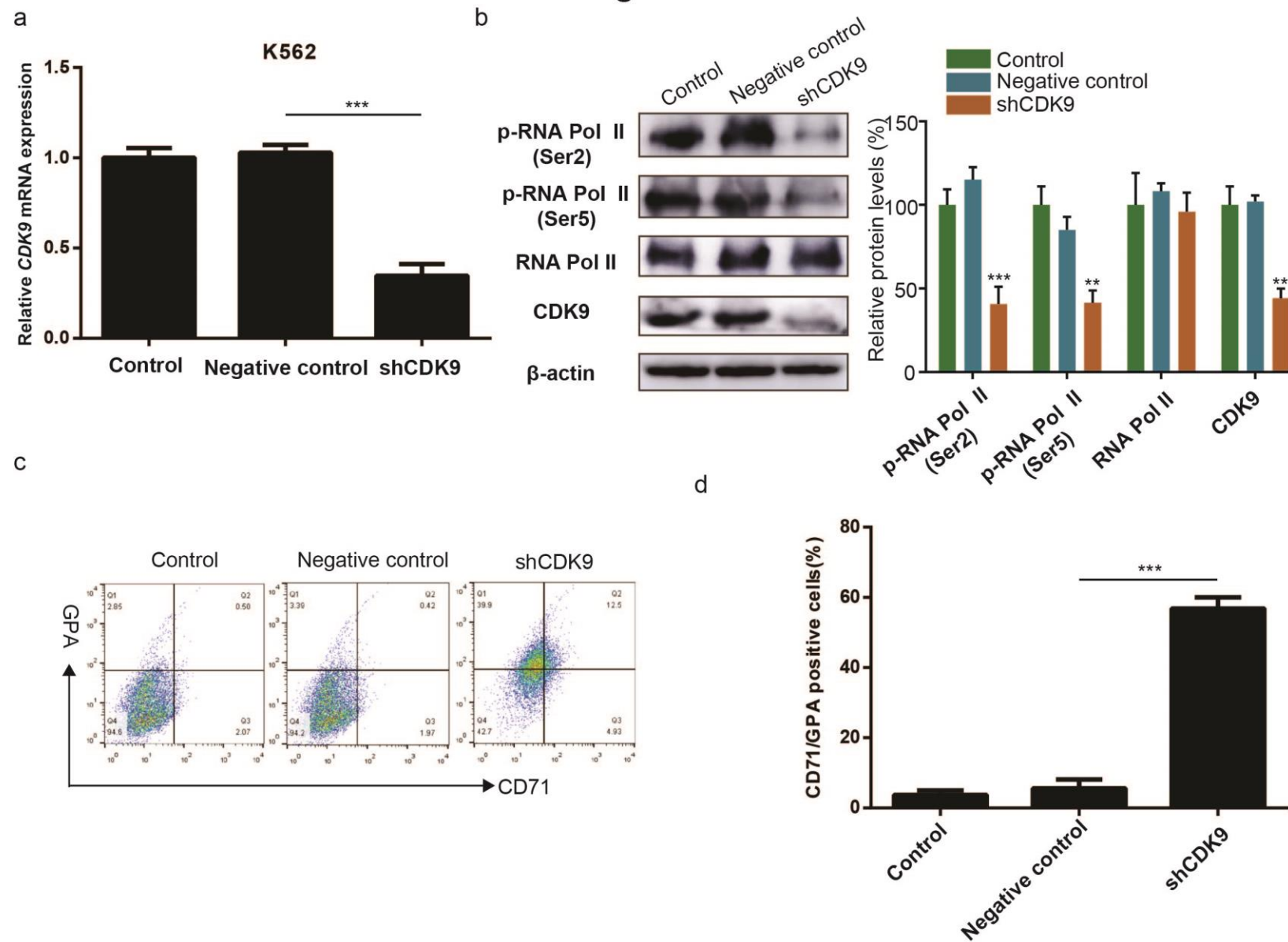

Supplement: Supplementary file 2 — Additional file 1. Figure S1. CDK9 shRNA K562 cells enhanced the rates of erythroid differentiation. (a) K562 cells were transfected with NC shRNA or CDK9 shRNA. The mRNA level of CDK9 was detected by RT-PCR. Data represent mean ± SD of three independent experiments. ***p < 0.001, compared with control group. (b) In control, NC and shCDK9 groups, cell lysates were analysed for p-RNA Pol II (Ser2), p-RNA Pol II (Ser5), RNA Pol II and CDK9 expression by western Blot. Data represent mean ± SD of three independent experiments. *p < 0.05, **p < 0.01, compared with NC group. (c) In control, NC and shCDK9 groups, the expression of CD71 and GPA was detected by flow cytometry analyses. Data represent the mean ± SD of three independent experiments. (d) Quantification of the expression of CD71/GPA. Data represent mean ± SD of three independent experiments. ***p < 0.001, compared with NC group. [file 12964_2021_764_MOESM1_ESM.pdf]

Figure S2

a

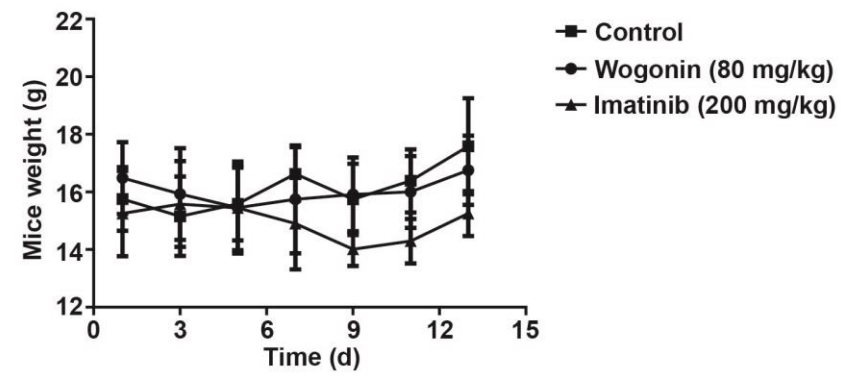

b

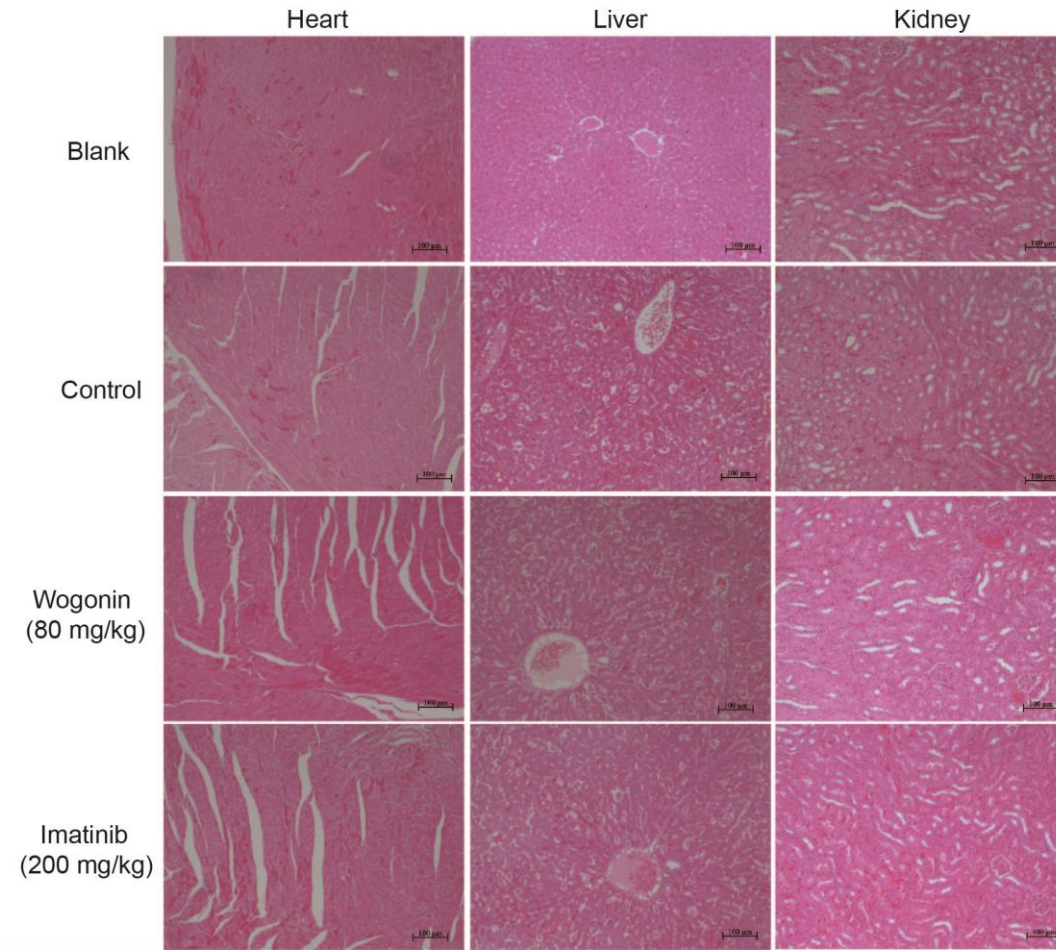

Supplement: Supplementary file 3 — Additional file 2. Figure S2. Toxicological assessment. (a) NOD/SCID mice weights were recorded every 2 days. (b) H&E stained main organs of mice from treated and control group to evaluate the toxicity of wogonin. [file 12964_2021_764_MOESM2_ESM.pdf]

Figure S3

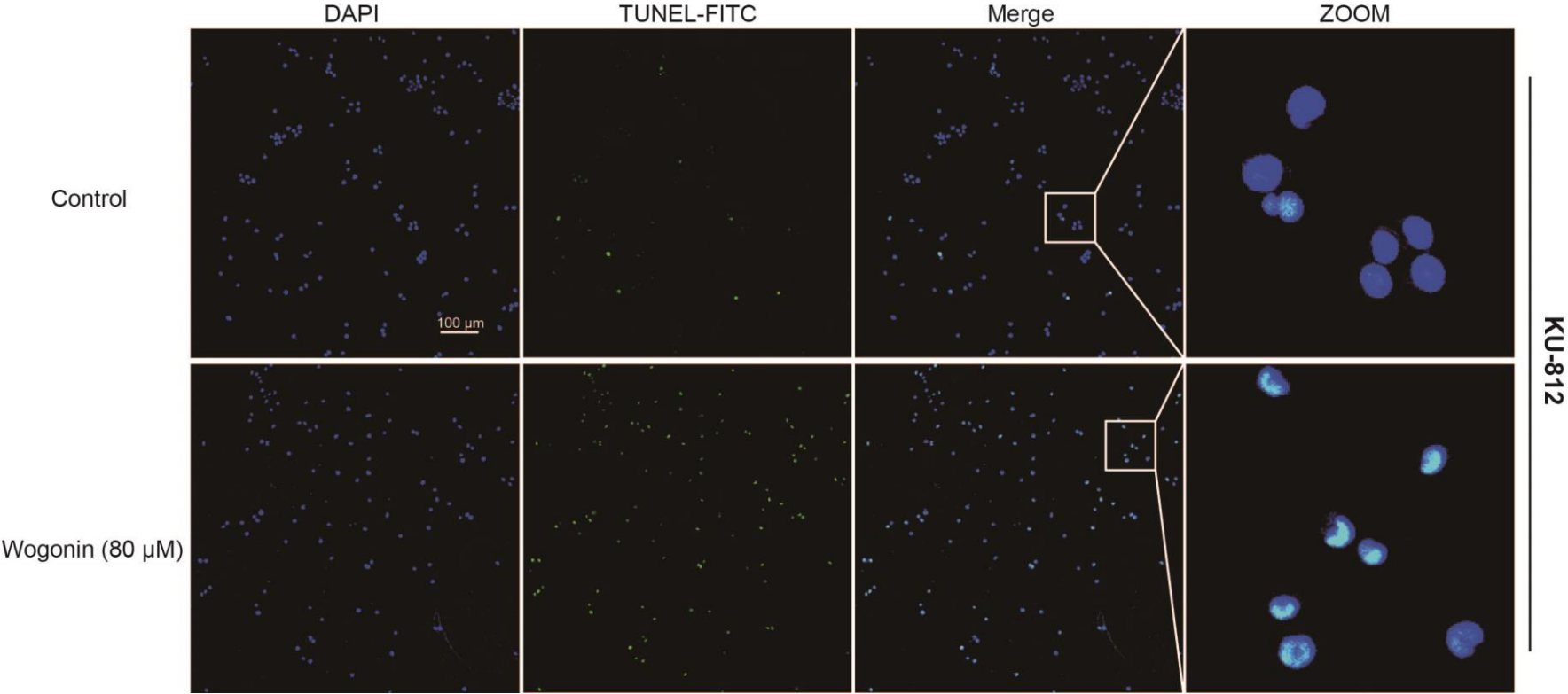

Supplement: Supplementary file 4 — Additional file 3. Figure S3. Apoptosis detection of KU-812 cells by TUNEL (a) KU-812 cells were treated with wogonin (0, 80 μM) for 48 h. Cell apoptosis was measured by TUNEL staining used a confocal microscope. Three visual fields were selected randomly for each specimen. [file 12964_2021_764_MOESM3_ESM.pdf]
